# Phosphatidylserine Counteracts the High Stocking Density-Induced Stress Response, Redox Imbalance and Immunosuppression in Fish *Megalobrama ambylsephala*

**DOI:** 10.3390/antiox13060644

**Published:** 2024-05-25

**Authors:** Yangyang Jiang, Zishang Liu, Ling Zhang, Wenbin Liu, Haiyang Li, Xiangfei Li

**Affiliations:** 1 Anhui Province Key Laboratory of Aquaculture and Stock Enhancement, Fisheries Research Institute, Anhui Academy of Agricultural Sciences, Hefei 230031, China; 2 Key Laboratory of Aquatic Nutrition and Feed Science of Jiangsu Province, College of Animal Science and Technology, Nanjing Agricultural University, No. 1 Weigang Road, Nanjing 210095, China

**Keywords:** stocking density, phosphatidylserine, stress response, antioxidant, fish culture

## Abstract

This study was conducted to investigate the effects of dietary phosphatidylserine (PS) supplementation on the growth performance, stress response, non-specific immunity and antioxidant capacity of juvenile blunt snout bream (*Megalobrama ambylcephala*) cultured under a high stocking density. A 2 × 2 two-factorial design was adopted, including two stocking densities (10 and 20 fish/m^3^) and two dietary PS levels (0 and 50 mg/kg). After the 12-week feeding trial, the high stocking density significantly decreased the final weight; weight gain rate; specific growth rate; feed intake; nitrogen retention efficiency; plasma complement 3 (C3) level; albumin/globulin (ALB/GLB, A/G) ratio; activity of myeloperoxidase, lysozyme (LZM) and glutathione peroxidase (GPX); *gpx* transcription; and abundance of sirtuin3 (Sirt3) and nuclear factor erythroid-2-related factor 2 (Nrf2). However, it significantly increased the plasma levels of cortisol, glucose (GLU), lactic acid (LD), total protein and GLB; hepatic malondialdehyde (MDA) content; and *sirt1* transcription. PS supplementation significantly increased the plasma ALB and C4 levels; the A/G ratio; the activity of LZM, CAT and GPX; the transcription of *sirt1*, *nrf2*, manganese-containing superoxide dismutase and catalase; and the Nrf2 abundance. However, it significantly decreased the plasma levels of cortisol, GLU and GLB, as well as the hepatic MDA content. In addition, there was a significant interaction between the stocking density and PS supplementation regarding the effects on the plasma LD, ALB, GLB and C3 levels; A/G ratio; hepatic CAT activity; and protein abundance of Sod2. In conclusion, PS supplementation can counteract the high stocking density-induced stress response, redox imbalance and immunosuppression in blunt snout bream.

## 1. Introduction

Recently, high-density intensive culture has become a common practice in modern aquaculture due to the increased demand for aquatic products and the pursuit of high profits [1]. However, high stocking densities can trigger a stress response in aquatic animals and compromise their health, thereby raising serious concerns about animal welfare. For example, water and air pollution problems are generally exacerbated under the high-density culture mode [2]. In addition, high-density culture can result in reduced growth performance, an altered body composition, a reduced antioxidant capacity and intestinal inflammation in fish [1,3,4,5,6]. This inevitably hinders the sustainable development of the aquaculture industry. Therefore, developing effective nutritional interventions to reduce the stress response and promote the health status of aquatic animals is crucial.

Phosphatidylserine (PS), also known as serine phospholipid or complex neuronic acid, is an active substance that constitutes the inner layer of biological cell membranes [7]. Previous studies have shown that PS is mainly found in brain cells and can (1) repair damaged nerve cell membranes and enhance the activity of nerve growth factors [8]; (2) change the fluidity of nerve cell membranes and increase the synthesis of acetylcholine [9]; and (3) reduce the levels of stress hormones like cortisol, thereby alleviating brain fatigue and mental stress [10]. To date, PS has been widely used as a nutritional supplement in various types of food and pharmaceuticals for sleep aids and the relief of low mood in humans [7,11]. However, its use in the aquaculture industry is still rarely reported. To the best of our knowledge, only one study has reported that PS can activate hemocyanin phenoloxidase activity to modulate the immune response in Atlantic horseshoe crab (*Limulus polyphemus*) [12]. However, whether it can alleviate the stress response in aquatic animals, thereby promoting their health, is still unknown.

Blunt snout bream (*Megalobrama ambylsephala*) is an herbivorous freshwater fish that is widely farmed in China due to its high survival rate, fast growth rate, disease resistance and flavorful meat, with annual production of 767,343 tons in 2022 [13,14]. Because of the increased demand for its products, high-density culture has become a common practice in the practical farming of this species [3]. This inevitably leads to a stress response in this species and a decreased antioxidant capacity and causes an inflammatory response [1,5]. Therefore, developing nutritional interventions to promote its health status is crucial. Considering this, the present experiment was designed to investigate the effects of dietary PS supplementation on the growth performance, stress response, non-specific immune function and antioxidant capabilities of blunt snout bream cultured under a high stocking density. The results can advance the development of effective nutritional interventions to ensure the welfare of aquatic species subjected to the high-density aquaculture mode.

## 2. Materials and Methods

### 2.1. Experimental Design, Fish and Feeding Trial

A 2 × 2 factorial design was employed in this study with two stocking densities (10 and 20 fish/m^3^) and two dietary levels (0 and 50 mg/kg) of PS designated. Accordingly, a total of 4 experimental groups were set up, comprising a normal-density (10 fish/m^3^) group without PS supplementation (ND), an ND group supplemented with 50 mg/kg PS (NDPS), a high-density (20 fish/m^3^) group without PS supplementation (HD) and a HD group supplemented with 50 mg/kg PS (HDPS). The stocking densities were designated following a previous study that defined 240 g/m^3^ fish as the high-density group of blunt snout bream at the start of the feeding trial [3]. The high-density group in this study was subjected to 380 g/m^3^, taking into consideration the initial weight of the fish. Therefore, they were cultured under the high stocking density during the whole feeding trial. The dietary PS dose referred to the intake amount (600 mg per day) of PS in humans recommended by the National Health Commission of the People’s Republic of China [15] and was adjusted taking into consideration the body weight and ration size of the fish. 

Blunt snout bream was procured from an Ezhou (Hubei, China) fish hatchery and was short-term bred in several flowing cages (4 × 3 × 3 m, length–width–height) located in an artificial earthen pond. During this period, the fish were fed with a commercial feed (no. 122, Shuaifeng Feed Co., Ltd., Nanjing, China) for domestication for 2 weeks. Then, 720 healthy fish (initial weight, 19.73 ± 0.17 g) were randomly assigned to 12 cages (2 × 1 × 1 m, length–width–height) located in an artificial earthen pond with 20 fish per cage in the ND group and 40 fish per cage in the HD group. Then, the fish were fed the experimental diets (Table 1) three times (7:30, 12:00, 16:30 h) a day to visual satiation during a 12-week culture trial. Each group was tested in triplicate. Before conducting this study, we checked the feed intake data of several previous studies [16,17] using blunt snout bream as the target species. Then, an average ration size throughout the feeding trial was obtained. Accordingly, the dietary PS dosage was designated. This allowed the fish to receive the targeted amounts of PS during the whole feeding trial without frequently monitoring their body weight, which may trigger the stress response in fish and is not conducive to their health. During the feeding trial, the fish were cultured under the following conditions: the pH was maintained between 7.1 and 7.3, the water temperature varied between 26 and 28 °C, the dissolved oxygen was kept between 5.0 and 6.0 mg/L, and the total ammonia nitrogen was kept under 0.04 mg/L. 

### 2.2. Sample Collection

The fish in each cage were numbered with their weights and lengths measured after a 24 h fast to determine the growth performance parameters. Next, 4 randomly chosen fish from each cage were anesthetized with 100 mg/L of tricaine methanesulfonate (Sigma, Saint Louis, MO, USA). Shortly after, blood samples were taken from the tail veins using disposable medical syringes. Following centrifugation (3000× *g* at 4 °C for 10 min), plasma samples were collected and kept at −20 °C for subsequent analysis. To determine the biometric parameters, samples of the liver, viscera, and intraperitoneal fat were obtained and weighed and then stored in liquid nitrogen for subsequent analysis. 

### 2.3. Analytical Procedures

#### 2.3.1. Growth Performance and Feed Utilization Formula


$$Survival\;rate\;(SR,\%)=100\times \frac{final\;number\;of\;fish\;in\;each\;cage}{initial\;number\;of\;fish\;in\;each\;cage}$$

$$Weight\;gain\;rate\;(WGR,\%)=100\times \frac{\left[final\;body\;weight\;(g)-initial\;body\;weight\;(g)\right]}{initial\;body\;weight\;(g)}$$

$$\mathrm{Specific}\;\mathrm{growth}\;\mathrm{rate}\;(\mathrm{SGR},\;\%/d)=100\;\times \;\frac{(\ln W_{t}-\;\ln W_{0})}{\mathrm{days}}$$

$$Feed\;intake\left(FI,g\;per\;fish\right)=\frac{total\;feed\;intake\;of\;each\;cage\;(g)}{total\;number\;of\;fish\;in\;each\;cage}$$

$$Feed\;conversion\;rate\;(FCR)=\frac{feed\;intake\;(g)}{weight\;gain\;(g)}$$

$$Protein\;efficiency\;ratio\;(PER)=\frac{weight\;gain\;(g)}{protein\;intake\;(g)}$$

$$Nitrogen/energy\;retention\;efficiency\;(NRE/ERE,\%)=100\times \frac{\left[(W_{t}\times C_{t})\;-\;(W_{0}\times C_{0})\right]}{C_{diet}\times feed\;intake\;(g)}$$

$$Hepatosomatic\;index\;(HSI,\%)=100\times \frac{liver\;weight\;(g)}{body\;weight\;(g)}$$

$$Condition\;factor\;(CF)=100\times \frac{body\;weight\left(g\right)}{body\;length\;(cm{)}^{3}}$$

In the formulas, W_0_ and W_t_ are the initial and final weights, C_0_ and C_t_ are the initial and final nutrient content in the body, and C_diet_ is the nutrient content in the diet.

#### 2.3.2. Proximate Composition Analysis

The proximate composition of the experimental diets was measured according to the AOAC [18]. To determine the moisture content, samples were dried at 105 °C until they reached a consistent weight. The crude protein content (nitrogen content × 6.25) was estimated using an automated Kjeldahl nitrogen instrument (FOSS KT260, Herisau, Switzerland) to measure the nitrogen concentration. By using an ether extraction method, a Soxhlet system (Soxtec System HT6, Tecator, Höganäs, Sweden) was utilized to measure the lipid content. The samples were burned for four hours at 550 °C to determine the amount of ash. Finally, the gross energy content was determined using a bomb calorimeter (PARR 1281, Parr Instrument Company, Moline, IL, USA). 

#### 2.3.3. Plasma Indicator Analysis

The plasma level of cortisol was measured following the protocols reported by Winberg and Lepage [19]. The glucose (GLU) level was measured using the glucose oxidase method [20]. The lactic acid (LD) level was determined by the method reported by Shuang, et al. [21]. The plasma total protein (TP) and albumin (ALB) content were both determined by the method reported by Li et al. [22]. By deducting the albumin values from the total protein, the globulin (GLB) content was determined. Moreover, by dividing the ALB values by the GLB values, the A/G ratio was computed. The plasma complement 3 (C3) and 4 (C4) levels were measured using an enzyme-linked immunosorbent assay method [23]. Aspartate aminotransferase (AST) and alanine aminotransferase (ALT) activity were both measured by the method reported by Yuan et al. [24]. Lysozyme (LZM) and myeloperoxidase (MPO) activity were both measured according to Zhang et al. [25]. The temperature of the LZM enzymatic reaction was 25 °C, and the substrate concentration was the concentration when the *Lysomicrococcus Substration* reached 0.65–0.75 under absorbance at 450 nm at 25 °C. The substrate of the MPO enzymatic reaction was 3% H_2_O_2_ and the enzymatic reaction temperature was 37 °C.

#### 2.3.4. Hepatic Antioxidant Analysis

Liver samples were prepared according to LYGREN and WAAGBØ [26]. The content of malondialdehyde (MDA) was measured using the procedures outlined by Satoh [27]. The activity of catalase (CAT), glutathione peroxidase (GPX), and superoxide dismutase (SOD) was measured by the method reported by Zhang et al. [28]. The soluble protein content of liver homogenates was measured using the technique reported by Bradford [29].

#### 2.3.5. Real-Time Quantitative PCR

Using an RNA purification kit (Invitrogen, Carlsbad, CA, USA), the total RNA from the liver was extracted. Then, the extracted RNA’s purity and concentration were evaluated using the absorbance at 260 and 280 nm, respectively. The RNA’s reverse transcription was performed using an RT-PCR kit (SYBR^®^ Prime Script^TM^, Accurate Biology, Changsha, China). Next, using ddH_2_O, the resulting cDNA was diluted to 10%. On a QuantStudio7 Flex Real-time PCR instrument (Thermo Fisher, Waltham, MA, USA), the polymerase chain reaction was carried out using the SYBR^®^ Green II fluorescence kit (Accurate Biology, Changsha, China). Next, using ddH_2_O, the resulting cDNA was diluted to 10%. Ten microliters of 2 × SYBR^®^ Green real-time PCR master mix (Accurate Biology, Changsha, China), 7.2 microliters of water treated with DEPC, two microliters of DNA, and 0.4 microliters of each forward and reverse primer, forming a total volume of 20 microliters, were included in the reaction system. One cycle at 95 °C for 30 s and forty cycles from 95 °C maintained for 5 s to 60 °C maintained for 30 s were used to perform the reaction. Melting curve analysis was carried out with warming to 95 °C for 15 s and from 95 °C to 60 °C for 1 min. The primers were designed and synthesized based on the available sequences (Table 2). Using the 2^−ΔΔCT^ approach [30], the transcription of the target genes was standardized by elongation factor 1 alpha, which served as a reference gene.

#### 2.3.6. Western Blotting Assay

Phenylmethanesulfonyl fluoride (PMSF, Cat#20104ES03, Yeasen Biotechnology, Shanghai, China) was mixed with the RIPA lysis buffer (Cat#20101ES60, Yeasen Biotechnology, Shanghai, China) to produce a protein lysate containing 1 mM of PMSF. Then, the liver tissue was homogenized at 4 °C using this lysate. After this, the protein concentrations in the supernatants were measured using a BCA kit (Cat#E112-01, Vazyme, Nanjing, China). Using the lysates mentioned above, the protein concentrations in the samples were normalized. The proteins in the lysate were separated using 4–20% precast protein plus gel (Cat#36250ES10, Yeasen Biotechnology Co., Ltd., Shanghai, China) electrophoresis prior to being deposited onto polyvinylidene fluoride membranes. The membranes were blocked for 15 min in a fast-blocking solution (Cat#36122ES60, Yeasen Biotechnology, Shanghai, China) and then were treated with primary antibodies overnight at 4 °C. Anti-beta-actin (42 KDa, 1:1000, 66009-1-Ig, Proteintech, Wuhan, China), anti-sirtuin 3 (Sirt3) (28 KDa, 1:2000, Proteintech, 10099-1-AP, Wuhan, China), anti-superoxide dismutase 2 (Sod2) (25 KDa, 1:1000, Proteintech, 24127-1-AP, Wuhan, China), and anti-nuclear factor erythroid-2-related factor 2 (Nrf2) (68 KDa, 1:2000, Proteintech, 16396-1-AP, Wuhan, China) were all used. After 3 TBST washes, the membranes were treated for 2 h with secondary antibodies (1:5000, BA1054, Boster, Wuhan, China). The immunoreactive bands were found using a high-sensitivity chemiluminescence kit (E412-01/02, Vazyme, Nanjing, China). The bands were visualized and quantified using the Image J (Image J 1.53t, Bethesda, MD, USA) software with beta-actin used to standardize the protein expression.

### 2.4. Statistical Analysis

Two-way ANOVA (SPSS 22.0, SPSS Inc., Chicago, IL, USA) was used to evaluate all data in order to identify any significant differences between the treatments in terms of the stocking density, the dietary PS supplement, and their interactions. If the interactive effects were significant (*p* < 0.05), one-way ANOVA was conducted for further analysis, accounting for the normality and chi-square of the data distribution. If significance (*p* < 0.05) was detected, the means were subsequently ordered using Tukey’s HSD multiple range test. All data are expressed as the mean ± S.E. (standard error of the mean).

## 3. Results

### 3.1. Growth Performance and Feed Utilization

As shown in Table 3, neither the culture density nor the PS supplementation affected the survival rate (SR), feed conversion ratio (FCR), hepatopancreas somatic index (HSI), condition factor (CF), protein efficiency ratio (PER), and energy retention efficiency (ERE) (*p* > 0.05). However, a high stocking density significantly reduced (*p* < 0.01) the final weight (FW), weight gain rate (WGR), specific growth rate (SGR), feed intake (FI), and nitrogen retention efficiency (NRE). 

### 3.2. Stress Response Indicators

**Figure 1:**
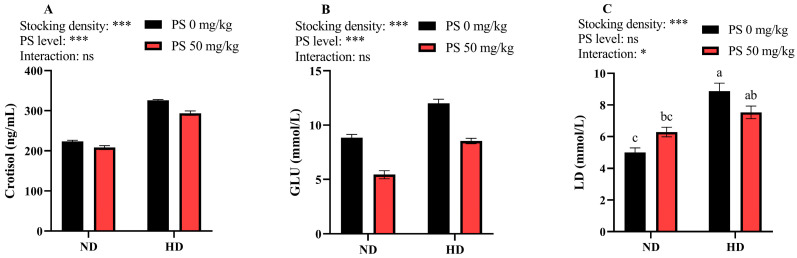
Plasma concentrations of cortisol (**A**), glucose (GLU, **B**), and lactic acid (LD, **C**) in juvenile blunt snout bream subjected to different stocking densities and phosphatidylserine supplementation. SD, stocking density; PS, phosphatidylserine; ND, normal density; HD, high density. Each datum represents the mean of four replicates. The two-way ANOVA result is indicated by asterisks. * *p* < 0.05, *** *p* < 0.001, ns: not significant. Significant differences (*p* < 0.05) between groups are indicated with different letters.

As shown in Figure 1, the high stocking density significantly elevated (*p* < 0.001) the plasma cortisol, GLU, and LD concentrations. However, PS supplementation significantly reduced (*p* < 0.001) the cortisol and GLU levels. In addition, a significant interactive effect (*p* < 0.05) between the stocking density and PS supplementation was noted for the plasma LD level.

### 3.3. Hepatic Injury and Non-Specific Immunity Indicators

**Figure 2:**
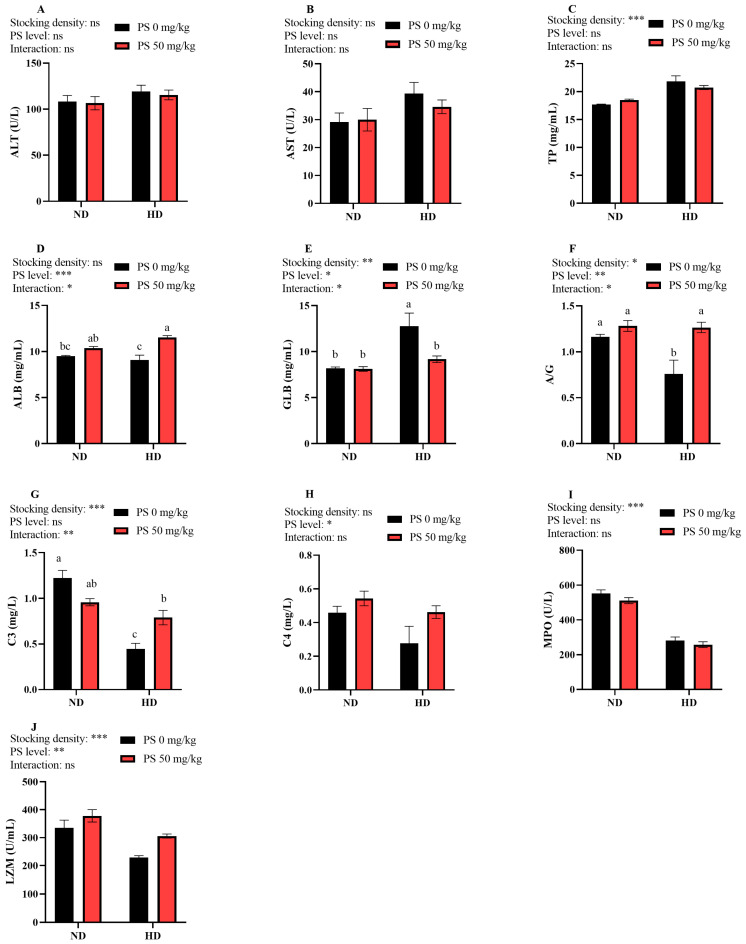
Plasma levels of total protein (TP, **C**), albumin (ALB, **D**), globulin (GLB, **E**), ALB/GLB (A/G, **F**), complement 3 (C3, **G**), and complement 4 (C4, **H**), as well as the activity of alanine aminotransferase (ALT, **A**), aspartate aminotransferase (AST, **B**), myeloperoxidase (MPO, **I**), and lysozyme (LZM, **J**), in juvenile blunt snout bream subjected to different stocking densities and phosphatidylserine supplementation. SD, stocking density; PS, phosphatidylserine; ND, normal density; HD, high density. Each datum represents the mean of four replicates. The two-way ANOVA result is indicated by asterisks. * *p* < 0.05, ** *p* < 0.01, *** *p* < 0.001, ns: not significant. Significant differences (*p* < 0.05) between groups are indicated with different letters.

Figure 2 shows that neither the culture density nor the PS supplementation affected the plasma ALT and AST activity (*p* > 0.05). The plasma TP and GLB concentrations increased significantly (*p* < 0.01) under the high-density culture, while the opposite result was observed in the plasma A/G ratio as well as the C3, MPO, and LZM activity (*p* < 0.05). In addition, PS supplementation significantly increased (*p* < 0.05) the plasma ALB and C4 levels, the A/G ratio, and the LZM activity, while it significantly (*p* < 0.05) decreased the GLB levels. Furthermore, the plasma ALB, GLB, and C3 levels, as well as the A/G ratio, were significantly (*p* < 0.05) affected by the interaction between the stocking density and PS supplementation.

### 3.4. Hepatic Antioxidant Indices

**Figure 3:**
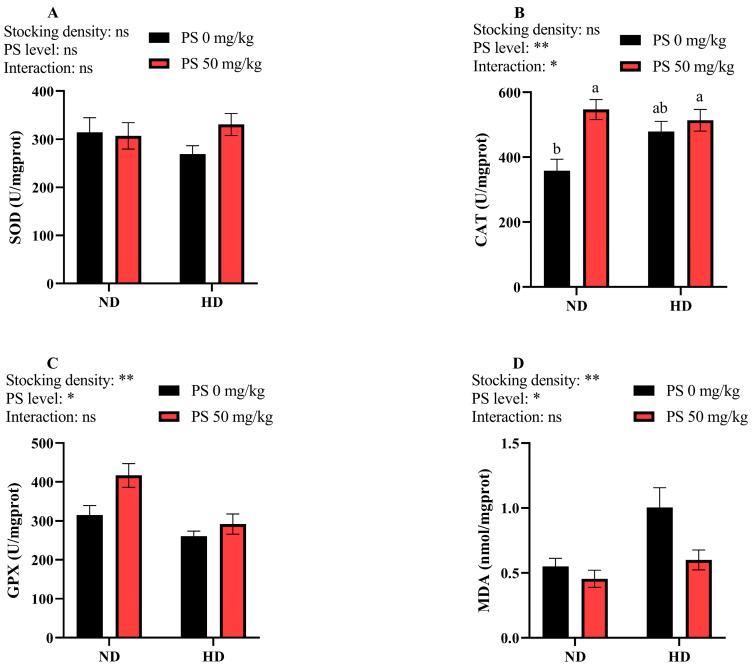
Hepatic activity of superoxide dismutase (SOD, **A**), catalase (CAT, **B**), and glutathione peroxidase (GPX, **C**), as well as the malondialdehyde (MDA, **D**) content, in blunt snout bream subjected to different stocking densities and phosphatidylserine supplementation. SD, stocking density; PS, phosphatidylserine; ND, normal density; HD, high density. Each datum represents the mean of four replicates. The two-way ANOVA result is indicated by asterisks. * *p* < 0.05, ** *p* < 0.01, ns: not significant. Significant differences (*p* < 0.05) between groups are indicated with different letters.

Figure 3 shows that neither the culture density nor the PS supplementation affected the hepatic SOD activity (*p* > 0.05). The high-density culture significantly (*p* < 0.01) reduced the hepatic GPX activity but elevated the MDA content. The dietary supplementation of PS significantly (*p* < 0.05) increased the liver CAT and GPX activity, but reduced the MDA content. Moreover, a significant interaction (*p* < 0.05) between the culture density and PS supplementation was noted for the hepatic CAT activity.

### 3.5. Expression Levels of Hepatic Antioxidant-Related Genes

**Figure 4:**
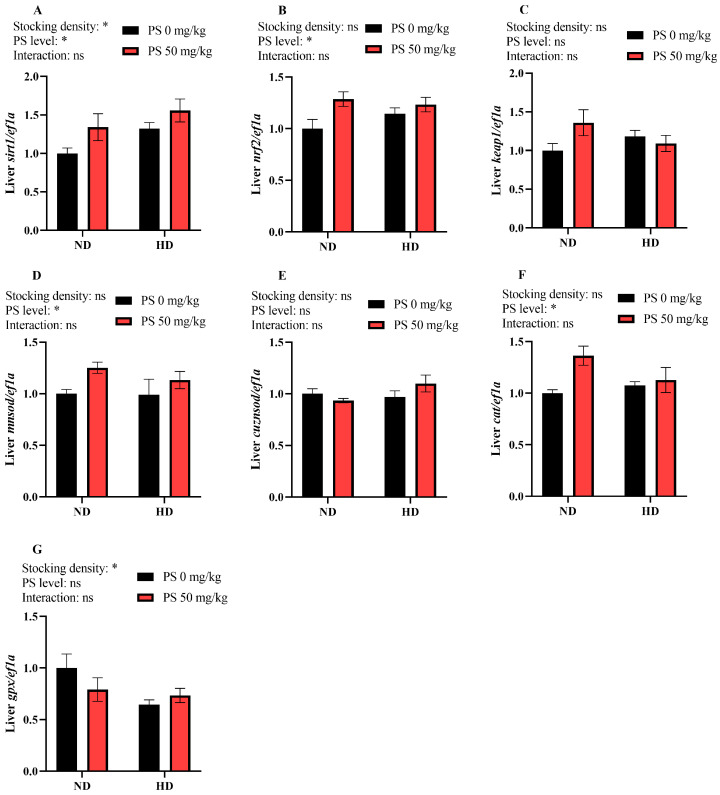
Hepatic transcription of sirtuin 1 (*sirt1*, **A**), nuclear factor erythroid-2-related factor 2 (*nrf2*, **B**), recombinant kelch-like ech-associated protein 1 (*keap1*, **C**), manganese superoxide dismutase (*mnsod,* **D**), copper-zinc superoxide dismutase (*cuznsod*, **E**), catalase (*cat*, **F**), and glutathione peroxidase (*gpx*, **G**) in blunt snout bream subjected to different stocking densities and phosphatidylserine supplementation. SD, stocking density; PS, phosphatidylserine; ND, normal density; HD, high density. Each datum represents the mean of four replicates. The two-way ANOVA result is indicated by asterisks. * *p* < 0.05, ns: not significant.

As shown in Figure 4, neither the culture density nor the PS supplementation affected the hepatic transcription of *keap1* and *cuznsod* (*p* > 0.05). The high stocking density significantly (*p* < 0.05) inhibited *gpx* expression but promoted *sirt1* expression. The dietary supplementation of PS significantly (*p* < 0.05) promoted the transcription of *sirt1*, *nrf2*, *mnsod*, and *cat*.

### 3.6. Expression Levels of Hepatic Antioxidant-Related Proteins

**Figure 5:**
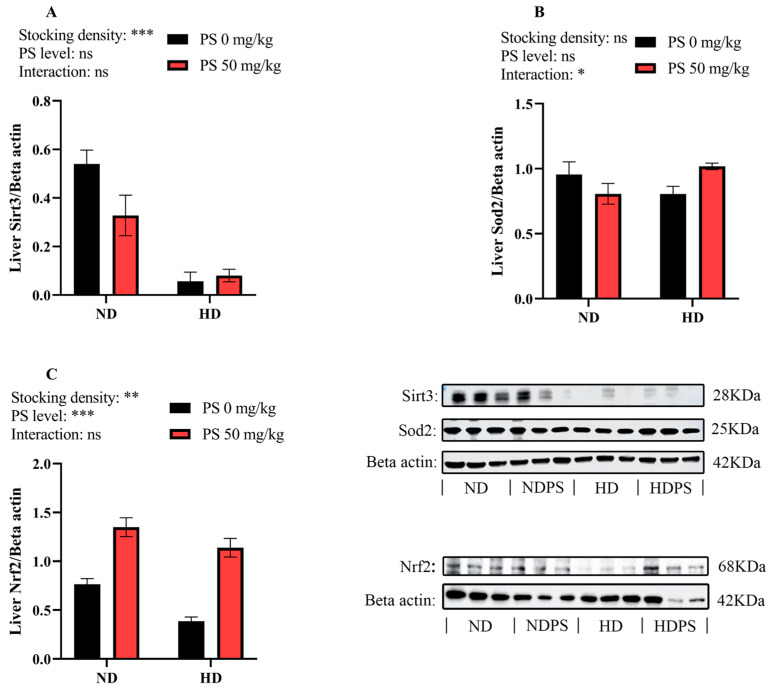
Hepatic protein expressions of sirtuin 3 (Sirt3, **A**), superoxide dismutase 2 (Sod2, **B**), and nuclear factor erythroid-2-related factor 2 (Nrf2, **C**) in blunt snout bream subjected to different stocking densities and phosphatidylserine supplementation. SD, stocking density; PS, phosphatidylserine; ND, normal density; NDPS, ND supplemented with 50 mg/kg PS; HD, high density; HDPS, HD supplemented with 50 mg/kg PS. Each datum represents the mean of three replicates. The two-way ANOVA result is indicated by asterisks. * *p* < 0.05, ** *p* < 0.01, *** *p* < 0.001, ns: not significant.

As shown in Figure 5, neither the culture density nor the PS supplementation affected the protein abundance of Sod2 (*p* > 0.05). However, the high stocking density significantly (*p* < 0.01) inhibited the protein abundance of Sirt3 and Nrf2, and PS significantly (*p* < 0.001) elevated the abundance of Nrf2. In addition, the stocking density and PS supplementation exerted a significant (*p* < 0.05) interactive effect on the Sod2 abundance, but no significance was observed among the four groups (*p* > 0.05). 

## 4. Discussion

In the present study, a high stocking density significantly reduced the FW, WGR, SGR, FI, and NRE of blunt snout bream, indicating that the growth performance and appetite of the fish were suppressed at a high stocking density. This result was parallel to those of previous studies on the same species [5,33]. The following reasons can be given for these results: (1) high-density aquaculture causes the competition of fish for food and space, which ultimately leads to a reduction in growth performance and food intake [3]; (2) a high stocking density can reduce the digestive enzyme activity of fish, thereby resulting in poor feed utilization and growth retardation [1]; and (3) high-density aquaculture can inhibit the expression of growth-related genes like growth hormones, growth hormone receptors, and insulin-like growth factor 1 in fish, thus reducing their growth rates [33]. However, neither the stocking density nor the PS supplementation affected the SR, FCR, HSI, CF, PER, and ERE of the fish, indicating that both the stocking density and PS administration had no significant influence on their survival and feed utilization. The following are the possible reasons for this: (1) when confronted with stimuli induced by the culture density, the stress response of fish enables them to maintain their homeostasis without disruption [34], and (2) the stocking density and PS supplementation in this study were not high enough to affect the survival and feed utilization of blunt snout bream. 

In the current study, the high stocking density markedly elevated the plasma cortisol, GLU and LD concentrations, suggesting that high-density culture causes a stress response in blunt snout bream. The following theories support this conclusion: (1) cortisol is generally released when fish are subjected to stressful conditions [34]; (2) elevated cortisol levels can enhance the gluconeogenic pathways of fish, thereby producing high amounts of GLU to provide sufficient energy to cope with stress [35]; and (3) anaerobic metabolism is enhanced in stressed fish, thereby leading to LD accumulation [36]. Moreover, stress reduces the appetite and feed intake of fish [37,38], which may account for the decrease in FI and feed utilization at high densities. However, the administration of PS markedly lowered the plasma cortisol and GLU levels, suggesting that PS can alleviate the stress response induced by crowding stress in blunt snout bream. This is not surprising, as a previous study has reported that the prolonged administration of PS counteracts the stress-induced activation of the hypothalamic–pituitary–adrenal axis in humans [39]. In addition, a significant interaction between the stocking density and PS supplementation was noted in the plasma LD levels, with PS elevating the LD level at a normal density but decreasing it at a high density. It is hypothesized that PS, as a nutraceutical, is metabolized through the liver, and the long-term administration of PS to healthy fish would increase the metabolic burden on the liver, which is also the site of LD metabolism [40]. However, at high-density conditions, PS can alleviate the stress response in fish, which in turn reduces LD levels [39].

In the present study, the high stocking density elevated the plasma activity of ALT and AST, although no significant difference was noted. This implies that the high-density aquaculture mode might lead to minor liver injury in blunt snout bream. In addition, PS supplementation in the ND group elevated the plasma AST activity, but it decreased in the HD group, confirming the conjecture about the metabolic burden on the liver caused by PS supplementation at a normal density. The high stocking density significantly decreased the plasma C3 levels and MPO and LZM activity, suggesting that the high-density culture reduced the non-specific immunity of blunt snout bream. It is widely acknowledged that (1) C3 is the most abundant component of the complement system, and its level can reflect the body’s immune capability [41]; (2) MPO is an enzyme involved in phagocytosis for bactericidal purposes [42]; and (3) LZM has antibacterial and antiviral effects by attacking the cell walls of bacteria for lysis, as well as inactivating viruses by binding directly to viral proteins [43]. However, the levels of TP and GLB showed opposite results, which might have been caused by the liver injury [44]. The decrease in the A/G ratio further supported this speculation. In addition, the dietary administration of PS markedly elevated the plasma ALB and C4 levels, the A/G ratio, and the LZM activity, suggesting that PS can enhance the non-specific immunity of fish. According to Coates, Kelly and Nairn [12], PS can enhance the immunity of Atlantic horseshoe crab (*Limulus polyphemus*) by activating hemocyanin. Furthermore, a significant interaction between the stocking density and PS supplementation was noted in the ALB, GLB, and C3 levels, as well as the A/G ratio. At a normal density, PS exerted no significant effect; however, PS significantly elevated these parameters at a high density. It was implied that the immune-enhancing role of PS might only be displayed under stressful conditions.

In this study, the high stocking density significantly reduced the GPX activity and elevated the MDA content, suggesting that the high-density aquaculture mode could reduce the antioxidant capacity of bunt snout bream. According to Yu, Yang, Liang, Ren, Ge, and Ji [4], the culture density can affect the mRNA expression of *gpx1* in this fish species, thereby influencing the redox defense. In addition, the administration of PS markedly enhanced the antioxidant capacity, as was revealed by the increased CAT and GPX activity and the decreased MDA content This result was expected, since PS has been reported to repair damaged cell membranes and improve the antioxidant capacity of the body [7,8,45]. In addition, there was a significant interaction between the stocking density and PS supplementation regarding CAT activity. PS significantly elevated the CAT activity at a normal density but exerted a limited effect at a high density. This difference is difficult to explain due to the fact that the relevant literature is lacking. 

To further unveil the molecular mechanisms underlying the antioxidant defense of fish, the transcription of the Keap1–Nrf2 pathway-related genes and proteins, as well as the abundance of the mitochondrial antioxidant Sirt3–Sod2 pathway-related proteins, was measured. The results showed that the high stocking density significantly elevated the *sirt1* transcription but decreased the *gpx* transcription and the protein levels of Sirt3 and Nrf2, suggesting that the fish were under oxidative stress when subjected to the high-density aquaculture mode, as *sirt1* is generally activated to reduce damage when animals are subjected to oxidative stress [46,47]. In addition, PS supplementation markedly increased the transcription of *sirt1*, *nrf2*, *mnsod*, and *cat* as well as the Nrf2 abundance, suggesting an enhancement in the antioxidant capacity. Previous studies have shown that under a normal state, Nrf2 binds to Keap1 and is inactivated; however, when the organism is subjected to oxidative stress, Nrf2 is released and activated by Sirt1 and subsequently binds to the antioxidant response elements, thereby exerting antioxidant effects by targeting several downstream effectors, including Mnsod [48,49]. Furthermore, an interactive effect between the stocking density and PS supplementation was noted in the protein abundance of Sod2, as was manifested by a decrease in Sod2 expression by PS at a normal density and an increase at a high density. This suggests that PS might only be effective in enhancing the mitochondrial antioxidant capabilities under stressful conditions.

## 5. Conclusions

In summary, the present study indicated that a high stocking density induced a stress response in blunt snout bream and reduced its growth performance, non-specific immunity, and antioxidant capacity. Dietary supplementation with 50 mg/kg PS can counteract these side effects caused by the high-density aquaculture mode. Based on this, PS has high potential to be used as a functional feed additive to promote the health status of fish cultured under high-density conditions, thereby improving the economic efficiency of intensive fish farming.

## Figures and Tables

**Table 1:** Formulation and proximate composition of the experimental diets.

Ingredients (%)	Diets (Phosphatidylserine Level, mg/kg)	
	0	50
Fish meal	5.00	5.00
Soybean meal	18.00	18.00
Rapeseed meal	20.00	20.00
Cottonseed meal	15.00	15.00
Fish oil	2.16	2.16
Soybean oil	2.16	2.16
Wheat flour	24.00	24.00
Wheat bran	4.50	4.50
Cellulose	5.99	5.99
Ca(H_2_PO_4_)_2_	1.80	1.80
Salt	0.40	0.40
Phosphatidylserine ^1^	0.00	50.00
Premix ^2^	1.00	1.00
Proximate composition (%)		
Moisture	8.98	8.87
Crude protein	30.49	30.51
Crude lipid	5.94	5.99
Ash	6.62	6.58
Gross energy (MJ/kg)	18.32	18.34

^1^ Phosphatidylserine (Cat#S832149-5g, Macklin, Shanghai, China) with purification of 50% was used in a double dose (100 mg/kg) to achieve the target concentration (50 mg/kg) in this study. ^2^ The premix supplied the following minerals (g/kg) and vitamins (IU or mg/kg): CuSO_4_·5H_2_O, 2.0 g; FeSO_4_·7H_2_O, 25 g; ZnSO_4_·7H_2_O, 22 g; MnSO_4_·4H_2_O, 7 g; Na_2_SeO_3_, 0.04 g; KI, 0.026 g; CoCl_2_·6H_2_O, 0.1 g; vitamin A, 900,000 IU; vitamin D, 200,000 IU; vitamin E, 4500 mg; vitamin K_3_, 220 mg; vitamin B_1_, 320 mg; vitamin B_2_, 1090 mg; vitamin B_5_, 2000 mg; vitamin B_6_, 500 mg; vitamin B_12_, 1.6 mg; vitamin C, 5000 mg; pantothenate, 1000 mg; folic acid, 165 mg; choline, 60,000 mg.

**Table 2:** Nucleotide sequences of primers used to assay gene expression by RT-PCR.

Gene Name	Forward and Reverse Primers (5′-3′)	Accession Number or Reference
*sirt1*	CAAACGACTCGGAGCCTCAC	MT518159.1
	GGTCTCGTCTTCCGAACTGG	
*nrf2*	CTTTGATGGATGCCTTCGGC	[31]
	TCTGGGTAACGGGTGAATGC	
*keap1*	TGAGGAGATCGGCTGCACTG	[31]
	TGGCAATGGGACAAGCTGAA	
*mnsod*	TGTTGGAGGCCATTAAGCGT	KF195932.1
	AAAGGGTCTTGGTTAGCGCA	
*cuznsod*	CACGCTCAACTTTGGCACAT	KF479046.1
	TGTCAACAGGGAGACCATGC	
*cat*	CCGGGGGATATCAGTTGGGT	KF378714.1
	TCCAAACCACTGAACTCGGG	
*gpx*	GAACGCCCACCCTCTGTTTG	[32]
	CGATGTCATTCCGGTTCACG	
*ef1a*	CTTCTCAGGCTGACTGTGC	X77689.1
	CCGCTAGCATTACCCTCC	

*sirt1*, sirtuin 1; *nrf2*, nuclear factor erythroid-2-related factor 2; *keap1*, recombinant kelch-like ech-associated protein 1; *mnsod*, manganese superoxide dismutase; *cuznsod*, copper-zinc superoxide dismutase; *cat*, catalase; *gpx*, glutathione peroxidase; *ef1a*, elongation factor 1 alpha.

**Table 3:** Growth performance and feed utilization of blunt snout bream subjected to different stocking densities and phosphatidylserine supplementation.

	IW (g)	FW (g)	SR (%)	WGR (%)	SGR (%/d)	FI (g)	FCR	PER	NRE (%)	ERE (%)	HSI (%)	CF
ND	19.73 ± 0.64	82.39 ± 3.04	96.67 ± 3.33	317.49 ± 7.41	1.70 ± 0.02	114.81 ± 7.52	1.84 ± 0.16	1.81 ± 0.15	38.33 ± 4.09	20.26 ± 2.67	1.23 ± 0.04	1.96 ± 0.03
NDPS	19.67 ± 0.41	94.44 ± 2.86	90.00 ± 5.77	380.01 ± 4.68	1.87 ± 0.01	128.06 ± 5.10	1.71 ± 0.13	1.92 ± 0.01	44.21 ± 2.53	22.07 ± 2.33	1.22 ± 0.02	2.09 ± 0.05
HD	19.60 ± 0.00	69.19 ± 1.65	91.67 ± 3.33	253.01 ± 8.42	1.50 ± 0.03	92.38 ± 1.25	1.87 ± 0.04	1.76 ± 0.04	32.73 ± 1.34	19.10 ± 1.82	1.26 ± 0.05	1.96 ± 0.06
HDPS	19.93 ± 0.26	70.27 ± 4.79	91.67 ± 6.01	253.23 ± 28.54	1.49 ± 0.09	97.91 ± 7.67	1.96 ± 0.11	1.68 ± 0.09	32.60 ± 3.24	16.48 ± 1.50	1.26 ± 0.04	1.97 ± 0.03
Two-way ANOVA												
SD	ns	***	ns	***	***	**	ns	ns	*	ns	ns	ns
PS	ns	ns	ns	ns	ns	ns	ns	ns	ns	ns	ns	ns
Interaction	ns	ns	ns	ns	ns	ns	ns	ns	ns	ns	ns	ns

SD, stocking density; PS, phosphatidylserine; ND, normal density; NDPS, ND supplemented with 50 mg/kg PS; HD, high density; HDPS, HD supplemented with 50 mg/kg PS; IW, initial weight; FW, final weight. The two-way ANOVA result is indicated by asterisks. * *p* < 0.05, ** *p* < 0.01, *** *p* < 0.001, ns: not significant.

## Data Availability

The data generated during the current study are available from the first author.
